# Clinical and immunological profile of patients with schizophrenia

**DOI:** 10.1192/j.eurpsy.2022.301

**Published:** 2022-09-01

**Authors:** N. Petrova, M. Mayorova, L. Churilov, A. Gvozdetskiy, I. Kudryavtsev, P. Sobolevskaia, V. Serazetdinova

**Affiliations:** 1 Saint-Petersburg State University, Department Of Psychiatry And Addiction, Saint-Petersburg, Russian Federation; 2 Saint Petersburg State University, Laboratory Of Mosaic Of Autoimmunity, Saint-Petersburg, Russian Federation; 3 North-western State Medical university named after I. I. Mechnikov, Chair Of Psychiatry And Addiction, Saint-Petersburg, Russian Federation; 4 FSBSI Institute of experimental medicine, Department Of Immunology, Saint-Petersburg, Russian Federation; 5 Saint-Petersburg FSBI Psychoneurological dispensary № 5, Day Hospital Department, Saint-Petersburg, Russian Federation

**Keywords:** schizophrénia, Cytokines, autoimmunity, lymphocytes subsets

## Abstract

**Introduction:**

The question of the involvement of inflammatory and autoimmune processes in schizophrenia pathogenesis has become the most relevant in the last decade and yet is not fully understood.

**Objectives:**

The study included 60 patients with paranoid schizophrenia (age 18 - 55 y.o.) and 30 healthy control group participants. Patients were in a stabilization state without a history of organic brain disorder or another verified somatic disease in the exacerbation phase.

**Methods:**

Research methods included follow-up method, neuropsychological (PANSS, BAC-S), laboratory (enzyme immunoassay, flow cytometry), and statistical.

**Results:**

Patients with schizophrenia had significant structural disorders of thinking, passive, apathetic withdrawal, negativism, impaired attention, psychomotor speed, volitional impulses. Cognitive impairment was detected in all study participants. Severe impairments are noted in the executive functioning, hand-eye coordination, attention, psychomotor speed. The severity of cognitive impairments correlated with the severity of clinical symptoms. Patients with schizophrenia had a significant decrease in central memory T-regulators levels, and an increase in Th1 and Th2 subsets, «double-positive» and «сlassic» Th17, Tfh2, «classic» Tfh17, and in Tfh17.1 (Pic.1).

**Figure:**
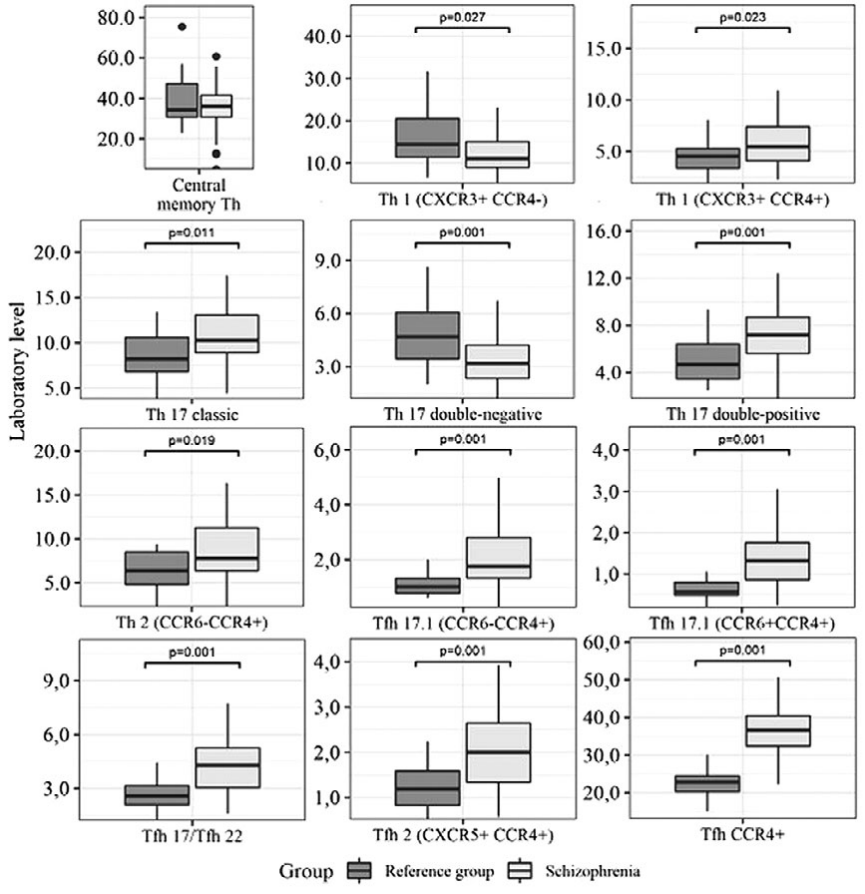

Picture. 1. T-helper subsets in patients with schizophrenia. They also had high levels of CCL20, IL-10, IL-12, IL-1β, IL-27, IL-31, IL-4, IL-13, IL-6, IL-9, TNFα in comparison with a control group. A significantly decreased levels of IL-17A, IL-17F, IL-2, IL-22, and TNFβ were also described in this group of patients.

**Conclusions:**

Patients with schizophrenia may be characterized by the presence of an inflammatory process and a high chance of autoimmunity. *Aknowledgement. This work was supported by the grant of the Russian Federation Government, contract 14.W03.31.0009*

**Disclosure:**

No significant relationships.

